# Who uses mental health support forums, and why? Triangulating findings from surveys, interviews, and forum posts

**DOI:** 10.1177/20552076261458957

**Published:** 2026-06-05

**Authors:** Zoe Glossop, Anna Lindroos Čermáková, Paul Marshall, Paul Rayson, Heather Robinson, Elena Semino, Fiona Lobban

**Affiliations:** 1 Spectrum Centre for Mental Health Research, Division of Health Research, 4396 Lancaster University, Lancaster, UK; 2 Linguistics and English Language, 4396 Lancaster University, Lancaster, UK; 3 School of Computing and Communications, 4396 Lancaster University, Lancaster, UK

**Keywords:** online forum, forum, mental health, online peer support, iPOF, mixed-methods

## Abstract

**Objective:**

Online forums for mental health, set up by charities, NHS services, and individual volunteers, have increased in popularity. Little is known, however, about who is using them and why. This study aimed to investigate this, using multiple methods to capture different types of users.

**Methods:**

A mixed-methods approach was used, with participants recruited from seven UK-based mental health forums as part of the Improving Peer Online Forums (iPOF) project. Forum users (n=791) participated in a survey which was compared to the demographics of NHS Talking Therapies. Twenty interviews with forum users were thematically analysed for reasons for use. Finally, the top keywords in the forums (a corpus of 28 million words) were calculated using the log likelihood test. One keyword was identified consistently across the forums, so its collocation profile was analysed.

**Results:**

Surveys showed the forums were predominantly used by white, female, and younger people, though there was a higher proportion of non-binary people compared to NHS Talking Therapies. Thematic analysis of interviews indicated that people used forums because they are easily accessible, overcoming barriers to in-person support, such as the need to speak. The linguistic analysis showed “scared” was a top keyword across all forums, with common collocations being I’m scared because and scared of. Reviewing posts showed users shared fears over mental health symptoms and identity.

**Conclusion:**

Forums are important alternative and complementary sources of mental health support, particularly for those facing discrimination or access barriers. Forums provide accessible, anonymous spaces for peer support, to share fears and connect with others.

## Introduction

Currently, most people in the United Kingdom (UK) experience barriers to accessing mental health services due to long waiting lists with the National Health Service (NHS), as well as stricter eligibility criteria for adult services.^1^ NHS Talking Therapies is a state-funded scheme which provides free therapy for people in the UK. Individuals can self-refer without needing a diagnosis or to see their general practitioner (GP). It was launched in 2008,^2^ and sixteen years on, the service faces immense pressure. In Southport (a UK region with the longest waiting times) people waited an average of 229 days from referral to first treatment with Talking Therapies in 2021/2022.^3^ Of the 1.81 million Talking Therapies referrals received across England in 2021/22, only 1.24 million entered treatment while 688,000 completed treatment.^3^ The high drop-out rate in mental health services is often because of dissatisfaction with long wait times, with people who tried to use the service reporting feeling “abandoned” and “hopeless”.^4^ In the current climate, where demand for mental health support far outpaces the NHS’s capacity,^5,6^ online forums could provide a more accessible support mechanism.

Increasing numbers of people are accessing online forums relating to mental health.^7,8^ For example, the subreddit r/mentalhealth grew from 20,000 members in 2016^9,10^ to 530,000 in mid-2025. Alongside this, health services have a growing interest in internet-based services, with online interventions being incorporated into policy.^11^ The UK National Health Service (NHS) website signposts adults with mental health problems to try online forums,^12^ while multiple NHS Talking Therapies services sub-contracted the online forum Togetherall (previously “Big White Wall”).^13–15^ Given the scale of NHS Talking Therapies, it may be useful to compare with the demographics of those using forums to assess whether online forums are serving populations already well-served by existing services, or whether they are attracting people who are less represented in NHS services.

Previous research suggests that people from stigmatized groups, who experience barriers to accessing in-person services, may use forums as an alternative source of support. For example, many women experiencing perinatal mental illness do not access health services^16^ because they fear judgement and the involvement of social services.^17^ Online forums can offer an anonymous space for peer support, which may in turn encourage people to access services.^18^ Similarly, people living in rural areas often experience limited access to mental health services, fear of stigmatization, and social isolation.^19^ Online forums can offer virtual spaces for people in rural areas to establish social connections and a sense of belonging,^20^ overcoming geographical isolation^21^ and building resilience.^22^

Previous research also suggests that young people are likely to seek help online.^23^ Young people are particularly affected by the inaccessibility of mental health services because they are likely to have higher mental health needs,^24^ and less likely to be able to afford private services.^25^ Furthermore, as “digital natives”,^26^ using online search engines to find information on health issues is often their first choice.^23^ A systematic review^27^ of 21 studies of young people’s use of online forums found that forums can provide a pragmatic resource for informational and emotional support. Other research also suggests that adolescents form the main userbase of forums to discuss eating disorders, self-harm, and suicide,^28–30^ which have been associated with both harmful and beneficial impacts.^31^

Overall, previous research has suggested a variety of people are using different forums for different reasons, including to form social connections,^21^ to find peer support for stigmatized experiences,^18^ and because of barriers to other mental health support.^20^ Prior research in this field has tended to recruit from a single forum and relied on a single data source.^32^ For example, Moore and Ayers (2017)^18^ used only interviews to research forum use by women with postnatal mental illness, and the studies reviewed by Hanley et al. (2019)^27^ were limited to young people. Some studies have moved beyond single-method, single-forum designs. For example, Steiner and Farmer (2024)^22^ used qualitative interviews to explore how online forums can help build personal resilience for rural Australian residents. Furthermore, Farmer et al. (2025)^33^ combined digital ethnographies and interviews to explore how forums’ value is co-created between forum providers and users. This work shows how online forums can promote health, and the importance of triangulating multiple data sources to develop a holistic understanding of forums. However, this work focused on specific populations and geographic contexts, rural Australia, which limits its transferability to a broader UK context. Therefore, the diversity of forums and users in the UK remains poorly understood.

To gain a holistic understanding of who uses forums and why across a range of UK-based mental health forums, it is necessary to triangulate findings across multiple types of data, each of which has methodological benefits and drawbacks.^34^ Surveys of forum users offer the opportunity for large scale and low-cost data collection but cannot offer the depth of understanding achievable through interviews. Both surveys and interviews offer a reflexive perspective from forum users, though findings may be influenced by demand characteristics, recall bias, or social desirability bias. However, both methods benefit from being able to include people who use forums but never post, who are estimated to make up approximately 90% of forum populations,^35^ who would be entirely invisible to linguistic analysis of posts alone. In contrast, corpus linguistic analysis of forum posts offers direct insights into what people are actually writing online. This approach, which has increased in use in forum research over recent years,^32^ involves identifying statistically distinctive patterns in language use through keyword analysis, where words that occur unusually frequently in comparison to a reference corpus are identified.^36^

A comprehensive mixed-methods approach, triangulating findings from surveys, interviews and corpus linguistic analysis, was used to explore who uses forums and why. Several UK-based mental health forums were chosen to represent a diverse range of forum providers operating alongside or within the UK health service. While previous work has explored this question with individual methods or within specific populations, this study aims to combine these three methodological perspectives across a diverse range of forums, offering a more complete picture than any single method or forum could provide. This study is part of the Improving Peer Online Forums (iPOF) project, which is a large interdisciplinary NIHR-funded realist-informed evaluation.^37^ Following a realist framework, the iPOF project aims to understand the underlying mechanisms that shape forum use: how forums work for different people across different contexts. A detailed protocol for the whole project is published.^37^ This study focused on the question “Who uses online forums for mental health support, and why?”, which was operationalized as the following research questions.

### Who uses online forums for mental health support?

• What are the demographics, (age, gender, ethnicity) of people who use different mental health forums?
• How do the demographics of those using forums differ from the demographics of people accessing NHS Talking Therapies?

### Why do people use online forums for mental health support?

• What reasons do people give for forum use in a survey?
• How do people explain their reasons for using forums when interviewed?
• What distinctive words and phrases do people use in forum posts, and what do these suggest about users’ motivation for engaging with online mental health forums?

## Methods

This study used a mixed-methods convergent parallel design, where three methods were conducted independently and the results brought together in the overall interpretation.^38^ Mixed-methods approaches are necessary to develop a holistic and comprehensive understanding of online health system use,^34,39^ including who uses mental health forums and why. The methods included descriptive analysis of survey data and comparison tests with routinely collected NHS data, thematic analysis of interviews, and corpus linguistic analysis of forum post data. The findings from each approach are considered together in the discussion and not related to the wider realist framework that was developed in the iPOF study,^37^ the results of which are reported elsewhere.^40,41^

### Ethics

Ethical approval for the iPOF project (including this study) was granted by Solihull Research Ethics Committee (IRAS 314029). All survey and interview participants provided informed consent to participate.

The collection of forum post data needs to be carefully considered due to ethical issues over anonymity, consent, the right for participants to withdraw, and the reasonable expectation of privacy for forum users.^42,43^ Considering these issues, we worked with forum hosts, users and moderators to develop an ethical framework for this study. For publicly open forums, consent was not required, but we posted information about the study to the forum with a designated email inviting questions and providing the opportunity to withdraw. For private forums where users could freely give consent during the sign-up process, only posts made by consenting users were included. For private forums where consent was not provided at sign-up, we asked the forum hosts to invite all users to take part by email which included a participant information sheet and consent form. Only posts made by consenting users were used. To protect our participants and forum partners’ anonymity, all participants were anonymized, and the forums have been pseudonymized using bird names. Additionally, all examples quoted from the forums are paraphrased so that they cannot be linked directly back to the forum or user.

A comprehensive account of the iPOF project’s ethical approach is published elsewhere.^44^

### Data sources - Forums

Forum owners were approached and included in iPOF if they were UK-based (though see Limitations on the geographical diversity of the forum participants) and were focused on mental health. A multiple case series design was used to recruit seven forums, purposively sampled for diversity across hosts, target populations, design (including level and nature of moderation, and whether they require registered login), and size. Forum hosts included an NHS Trust, mental health-related charities, companies, as well as individual moderators who had created mental health-specific forums within larger public platforms. Seven forums were represented across two methods of research (surveys and interviews), while the linguistic analysis of the posts was based on three forums (see details below). A brief description of each forum and the type of data analyzed from each is summarized in Table 1. A full description of each forum involved in the study can be read here: https://www.lancaster.ac.uk/ipof/case-summaries.

**Table 1.** Brief description of each forum and the data collected.

Forum	Description of forum	Survey N	Interview N	Language data N		
				Words	Posts	Users
Sparrow	Private forum with subforums to discuss different mental health issues.	107	3	n/a	n/a	n/a
Robin	Private forum for people with bipolar disorder and carers.	12	2	n/a	n/a	n/a
Magpie 1,2,3 4,	Public forum with subforums ran by charities for different health issues. Four subforums were included in the study: Magpie.1 is about mental health issues generally; Magpie.2 provides resources and a place for discussion for people with eating disorders; Magpie.3 is for people with post-natal illness, and Magpie.4 is for people with postpartum psychosis.	110	4	9,801,121	99,520	7,517
Dunnock	Private forum for young people to discuss mental health.	287	5	13,425,212	213,125	42,648
Jay	Private forum for adults to discuss mental health.	13	2	n/a	n/a	n/a
Chaffinch	Public forum for young people to discuss mental health.	49	1	n/a	n/a	n/a
Starling	Public forum to discuss mental health generally.	213	3	4,820,253	46,868	6,258
Total	​	791	20	28,048,586	359,513	56,423

### Survey

#### Recruitment

Participants were recruited from seven online mental health forums, using advertisements posted to the forum and, when possible, through direct emailing to registered forum users. Users were emailed by the forum hosts, with ethically-approved wording that was provided by the research team. Users were emailed on the addresses associated with their forum account only if they had previously consented to receiving research-related emails from the forum platform. Participants were invited to take part in the survey if they were a) over 16 years old, b) a UK resident, and c) had visited the forum at least once before. These were assessed by self-report questions. After confirming that they met the inclusion criteria, participants were emailed a unique link to complete the survey online. Participants received a £10 digital voucher upon completion.

The final dataset consisted of 791 survey participants, from seven forums.

#### Materials

The online survey was hosted on the Research Electronic Data Capture system (REDCap^45^). The design of the iPOF survey was guided by principles proposed by Westhorop and Feeny^46^ to test realist program theories. In the context of the wider iPOF study, the survey’s purpose was to test realist program theories and to sample participants for interview.^44^ The survey was co-designed by statisticians, clinicians, forum hosts, moderators and users in our public and patient involvement (PPI) group. Data was stored on REDCap during recruitment. After recruitment was completed, the data was downloaded without identifying fields and stored on Lancaster University’s OneDrive. The full survey data and details of its development and data management are published elsewhere.^44,47^ Participants completed the survey in an average of 24 minutes.

For the current study, to address who uses forums and why, a subset of 4 questions on demographics and reasons for forum use were used. Demographics (age, gender, ethnicity) were measured using categorical response options. The reasons for joining the forum were recorded with multiple response options. The question on reason for forum use is displayed in Textbox 1, while all survey questions used in the current study can be found in Supporting Information 1.

**Textbox 1. Survey question on the reasons for forum use**

“Thinking about the **first time** you ever came to this forum, which options best describe **the main reason** for your visit?” (pick more than one if you like).

0 “I wanted to find help, advice, information, or support for **myself**”
1 “I wanted to find help, advice, information, or support for **someone else** (e.g. friends, family)**”**
2 “I wanted to offer help, advice, support, or information to **other forum users**”
3 “Other reasons (please write in)”.

#### Comparison data: Talking therapies

To explore how the demographics of people using online forums for mental health support compared with people accessing NHS primary care, NHS Talking Therapies datasets were used. Demographic data from the 1,215,329 people accessing NHS Talking Therapies between 2022-23 was used to compare with the survey’s demographics. All data was sourced from the publicly available NHS Talking Therapies Annual Report 2022-23.^48^

#### Statistical analysis

Data management and analysis were conducted using Microsoft Excel^49^ and SPSS (Version 29).

The frequencies were reported for the age, gender, and ethnicity of participants from each of the seven forums. The survey results were re-coded to ensure comparability with Talking Therapies. For example, the survey recorded age categorically with 9-year buckets (25-34; 35-44, etc.) which were recoded to “25-64”, as reported in the NHS Talking Therapies data. Full detail of this is in Supporting Information 1. Chi-squared comparison tests were selected to analyze differences between the two groups because the dependent variables (age, ethnicity and gender) were nominal.^50^ Because the number of participants recruited from each forum was too small to make statistically valid comparisons between them, the survey data from the 791 participants from across the seven forums was pooled into one forum-user group.

### Interviews

#### Recruitment

A convenience sample of twenty forum users were recruited through forum advertisements and through expressions of interest on the survey. The research team shared the participant information sheet and topic guide over email with potential participants, and participants gave consent using an online forum hosted on Qualtrics. Interviews could take up to an hour, and participants received a £30 digital voucher as a thank-you for their time.

#### Materials

Interviews were conducted remotely, using Microsoft Teams for video calls or phone calls depending on the participant’s preference. Interviews were recorded using an encrypted voice recorder and the recording was deleted after transcription. Transcription was conducted by an independent transcriber. Semi-structured realist-informed interviews were conducted by PM and ZG. The topic guide began with openly exploring participants’ experiences of using forums, before progressing to more specific questions to test theories on how online forums work.^51^ The topic guide was co-designed with clinicians and the PPI group. An earlier iteration of the guide was used for practice interviews with forum users in the PPI group, who provided feedback to refine the topic guide. The full topic guide is available in Supporting Information 2.

The analysis for this study focused on participants’ responses to the first section of open-ended questions, including “what motivated you to start using it?”. This was because the rest of the interview focused on specific theory-testing questions such as on forum design and safety. A reflexive thematic analysis, following the guidance of Braun and Clarke^52^ was conducted, exploring why participants used the forums. The analysis process began with data familiarization; ZG read all twenty transcripts. Interviews were coded inductively by ZG for reasons for forum use, using NVivo.^53^ Preliminary coding and themes were discussed by ZG, FL and PM. After these discussions, ZG refined the coding and thematic structure, and a consensus on thematic structure was reached after review and discussion in a meeting with ZG, FL, PM, ES, and PR.

### Linguistic analysis of the forum posts

#### Data collection

The language data was collected from three forums, Starling, Dunnock, and Magpie between March 2016 and March 2024. Magpie included four subforums, which were kept separate for this analysis. Starling data was collected by the research team using the platform’s application programming interface (API). Dunnock and Magpie data was shared with the research team by the forum hosts.

The three forums were selected to represent different user demographics and different hosts, which ensured a diverse range of contexts were included, while also keeping the full dataset a manageable size by not including every forum.

#### Analysis

We used a combination of both quantitative and qualitative methods to identify linguistic patterns that are relevant to our research questions. As our research questions were broad, we first adopted a well-established corpus linguistic technique known as ‘keyness’ analysis. This approach has been used in health and online forum research previously.^54,55^ This involves comparing the relative frequencies of words in a ‘target’ corpus with the relative frequencies of words in a ‘reference’ corpus. The output is a list of words, known as ‘keywords’ that are statistically significantly more frequent in the target corpus than in the reference corpus. In our analysis, each of the six forums mentioned above was treated as a target corpus: Starling, Dunnock and the four Magpie forums. A corpus sampled from the written subcomponent of the 1994 version of the British National Corpus (BNC) was used as a reference corpus.^56^ The BNC is the standard corpus used in linguistic studies as it is considered broadly representative of general language use.^57^ The written component was selected for comparability with written forum posts. We used the log likelihood measure of statistical significance^58^ to calculate and rank the keywords from each of the forums. We compared the top ten keywords in each of the target corpora (six keyword lists altogether) and their log likelihood values. In the comparison, we aimed to identify keywords that occurred consistently across the top ten keywords in all the keyword lists. The comparison showed there was only one such keyword (*scared*) and this was further analyzed through its collocation profile. In corpus linguistics, collocates are words that co-occur in close vicinity next to each other in a statistically significant way and they reveal important information on the word’s linguistic usage.^59^ The collocation analysis was performed in AntConc software.^60^ To calculate the collocations, we used likelihood statistics and a span of five words to the left and right of the keyword. Our dataset is, in terms of its size, heavily skewed towards posts retrieved from Dunnock – 48% of the total word count, while posts from Starling take up only 17% of the overall wordcount. For the collocation analysis, we have therefore added an additional condition for the collocate to occur in all three forums to be considered for detailed analysis.

## Results

This section will cover the results from the survey first, the interviews second, and finally the linguistic analysis of forum posts.

### Survey

Across all the forums, most survey participants were white and female, and the most common age bracket was 16-24. The full demographics are reported in Table 2.

**Table 2.** Demographics of the forum survey, n=791.

​	N	%
Age (years)		
16-24	285	36
25-34	196	24.8
35-44	133	16.8
45-54	68	8.6
55-64	65	8.2
65+	41	5.1
Prefer not to say	3	0.3​
**Gender**		
Female	474	59.9
Male	253	31.9
Non-binary	46	5.3
Prefer not to say	13	1.6
Prefer to self-describe	5	0.6
**Ethnicity**		
White	643	81.3
Black	37	4.7
Asian	35	4.4
Mixed	55	6.9
Prefer not to say	13	1.6
Prefer to self-describe	8	1.01

#### Comparison with talking therapies

Table 3 shows the comparison between the survey group and the Talking Therapies group. Significant differences were found across each of the sociodemographic variables, although the effect sizes were weak.^61^ The comparison suggests a greater proportion of young people aged 16 – 25 accessing online forums, compared to people accessing Talking Therapies. There was a smaller proportion of females accessing online forums compared with Talking Therapies, despite females being the dominant gender across both datasets. There was a larger proportion of the White and Mixed ethnic groups accessing online forums compared with Talking Therapies, although the White group was the majority across both datasets.

**Table 3.** Chi-squared comparisons between forum and Talking Therapies samples.

​	iPOF forums n=791		Talking therapies n=1,215,329		Chi-squared test
	%	95% CI	%	95% CI	
**Age (Years)**					χ2(3)=4703.21, *p*<.001, Cramer’s V=0.062
Under 25	36.0	(32.7 – 39.4)	21.9	(21.8 – 22.0)	
25 – 64	58.4	(55.0 – 61.8)	71.3	(71.3 – 71.4)	
65 and over	5.2	(3.6 – 6.7)	6.8	(6.7 – 6.8)	
Not stated	0.4	(0.0 – 0.8)	0.0	0	
**Gender**					χ2(3)=856.00, p<.001, Cramer’s V=0.027
Male	32.0	(28.7 – 35.2)	31.8	(31.7 – 31.8)	
Female	59.9	(56.5 – 63.3)	66.5	(66.4 – 66.6)	
Indeterminate	6.5	(4.7 – 8.2)	0.3	(0.3 – 0.4)	
Not stated	1.6	(0.7 – 2.5)	1.4	(1.3 – 1.4)	
**Ethnicity**					χ2(5)=84.19, p<.001, Cramer’s V=0.008
Asian	4.4	(3.0 – 5.9)	6.8	(6.8 – 6.9)	
Black	4.7	(3.2 – 6.2)	4.1	(4.1 – 4.1)	
Mixed	7.0	(5.1 – 8.7)	3.2	(3.2 – 3.3)	
White	81.3	(78.6 – 84.0)	76.2	(76.2 – 76.3)	
Other Ethnic Group	1.0	(0.3 – 1.7)	2.3	(2.3 – 2.4)	
Not stated	1.6	(0.8 – 2.5)	7.3	(7.2 – 7.3)	

#### Breakdown across the forums

**Figure 1.**
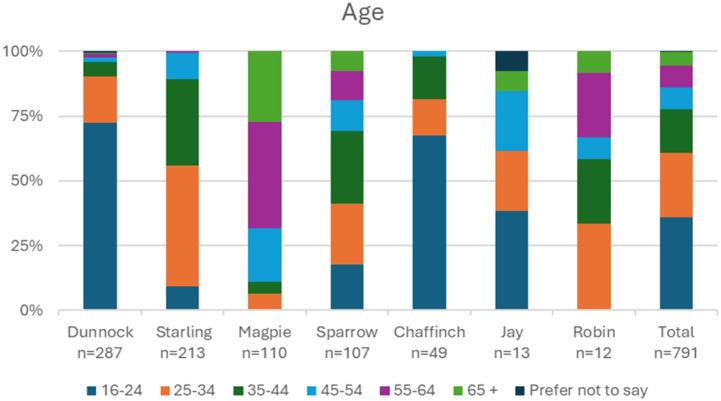
Breakdown of age reported on survey.

**Figure 2.**
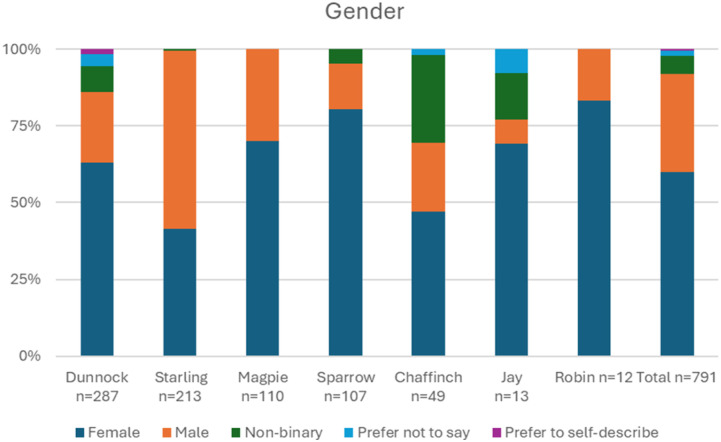
Breakdown of gender reported on survey.

**Figure 3.**
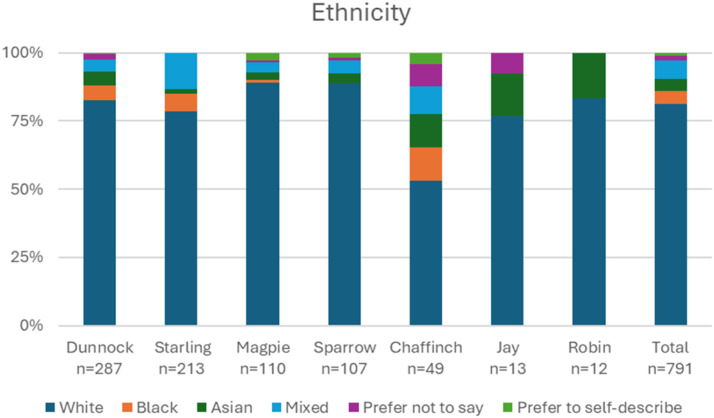
Breakdown of ethnicity reported on survey.

There was some variation across the different forums in the participant demographics, displayed in Figures 1–3. Dunnock and Chaffinch (two forums aimed at young people) had the highest proportions of young participants, with 72% (241) of their participants in the 16-24 bracket. In contrast, 89% (98) of Magpie (a public platform with charity-run sub-forums) participants were aged 45 and over. Sparrow (an NHS based forum) had a slight majority (28%, n=30) of participants in the 35-44 bracket, although there was representation of participants across all age brackets. Participants from 6 out of 7 forums were majority female. In Starling (a public forum to discuss mental health generally), the majority was male. Chaffinch showed the highest proportion of non-binary participants (22%, 11), which was the second most frequently reported category after female. Participants were mostly White across all forums.

#### Reasons for use

Table 4 shows the proportion of reasons that respondents gave for visiting the forum. Most participants (69%) reported wanting to find support for themselves while 29% visited the forum to support other forum members.

**Table 4.** Reason for visiting forum.

​	N (%)
I wanted to find help, advice, information, or support for **myself**	544 (69%)
I wanted to find help, advice, information, or support for **someone else** (e.g. friends, family)	237 (30%)
I wanted to offer help, advice, support, or information to **other forum users**	229 (29%)
Other reasons	20 (3%)

Twenty participants mentioned other reasons, including wanting “to vent to others”, wanting “to see if other people had been through similar things so I didn’t feel alone”, as well as because the forum was recommended to them by others.

### Thematic analysis of interviews

The sample of 20 participants were mainly White/White British, female and in the 16-25 age bracket. Full demographic data is displayed in Table 5.

**Table 5.** Demographics of interview participants (n=20).

​	**N**
**Age (Years)**	
16-25	7
26-35	4
36-45	2
46-55	3
56-65	2
66-75	2
**Gender**	
Female	15
Male	3
Non-binary	1
Prefer not to say	1
**Ethnicity**	
Asian/Asian British	3
Black/African/Caribbean/Black British	2
White/White British	15

Thematic analysis of the interviews identified five themes describing why participants used online mental health forums. These were accessibility, a preference for typing, as a last resort after trying other options, to form social connections, and to give back to the community. Each theme is described in detail below, including illustrative quotes. Additional quotes are available in Supporting Information 3.

#### Anytime, anywhere: The value of accessibility

Participants described forums as highly accessible, which was a key reason for their forum use. Important features of accessibility were the forum being publicly available, having search functions, being easy to set up and access across multiple devices, and being available 24/7.

Firstly, account creation as described as quick and straightforward, and the ability to access forums with a mobile phone was important for day-to-day use.

“*You can make an account very quickly and you can just join in just like that*”. Chaffinch user.

“*If it wasn’t on my phone I don’t think I’d use it. Literally I’m permanently logged in on the memory – so I just type [Sparrow] and it comes up with the username and log in … I don’t think I would specifically open my laptop to go on it*”. Sparrow user.

After registering, participants described how the ability to search existing posts for relevant experiences was often preferred to creating an original post.

“*if I’m having issues then I’m not sure but I might want to actually see if there’s any posts on it beforehand – I prefer not to actually have to ask or create my posts. I prefer to do that and yeah so try and look through if there’s any posts similar to my issue so yeah I just kind of look through those*”. Starling user.

The 24/7 availability of forums was especially important, allowing participants to access support at any time, including in between more structured support such as therapy sessions. For example, one participant described their therapist recommending the forum for use between appointments.

*“it was advised – or it was offered to me by a cognitive behaviour therapist counsellor as an extra resource in the week when I was speaking to her”*. Sparrow user.

The 24/7 availability was also important for participants who struggled at night, often due to mental health issues. For example, one participant described how Robin had a “can’t sleep” thread, allowing users to support each other when awake at night.

*“Sometimes you feel like it’s dark and everybody is asleep and you’re just alone suffering by yourself but being able to access the forum and this particular thread it makes you feel a bit nicer”*. Robin user.

Forums were contrasted with and preferred over other 24/7 options for mental health support such as helplines or one-to-one webchats. These often had waiting times or were targeted to people experiencing acute distress, such as feeling suicidal, so forums were more appropriate for lower-level distress.

“*You can call Samaritans at three in the morning but you might not want to. We do often have users and I’ll be on late at night if I’m not sleeping so you can kind of get 24 hour support… You don’t have to leave the house to access the forum. You don’t have to talk to anyone”.* Starling user.

However, participants also described how the ability to access forums at any time could be detrimental. For example, unrestricted access to a forum about anxiety could contribute to a person’s rumination and ultimately worsen their anxiety.

*“I feel like I reflect more on my own anxiety and I sort of increase my anxiety because of that so I think that’s probably where my problem lies with that sort of community… To be honest, I think the only one [forum] that would probably have a negative impact on my mental health is a mental health forum.”*. Magpie user.

#### “Happier to type”: In your own time, space and words

Participants described a preference for the written, asynchronous nature of forum communication as a distinct reason for using forums. Typing and reading, rather than speaking, created a sense of psychological distance and safety that participants found easier to manage when discussing difficult experiences.

The asynchronous nature of forums meant there was no pressure to respond in real-time to other users.

“*it’s not like meeting somebody or phoning somebody where you have to have an immediate answer”*. Sparrow user.

This was important for participants with spoken communication difficulties, such as being deaf, being neurodivergent, or having severe anxiety. Being able to read and respond in their own time, without the chance of mishearing or the pressure of immediate verbal exchanges, made it possible for these participants to be supported in ways that other forms of support did not allow.

*“it’s so much easier to be able to just read and type*… *it’s all just texting and reading regardless of whether you have issues with hearing, lipreading or anxiety with speaking when you’re really distressed or autism*”. Jay user.

Several participants described previously having tried verbal-based support such as helplines, talking therapies, or support groups, but found the requirement to speak to be a barrier. People with mental health issues could struggle to verbally speak about their issues, especially if they were in a state of acute distress.

“*I had counselling and stuff before but I wasn’t – it sounds really weird but I wasn’t in a position where I wanted to talk. I was happier to type, so I needed somewhere I could go where I didn’t have to verbalize what was going on, I could write it down*.” Dunnock user.

“*I wouldn’t imagine many people whether they’re autistic or not when they’re crying or shaking when they’re really stressed actually wanting to talk talk”* Jay user.

For some younger participants, the preference for written communication was linked to their experience of the lockdown-accelerated shift to online communication. Online communication was the norm for this group, and face-to-face communication felt more uncomfortable.

“*Lockdown had a huge impact especially on my generation I’d say…because we’re all so used to talking online. I think when I went back into school I found it especially stressful. Talking to people face to face again was weird… I think that deterred me from talking to people as well after lockdown the fact that I’d just grown used to it online*”. Dunnock user.

Participants also described typing as a reflective and cathartic process, like journalling. Taking time to write and reflect helped participants to externalize their own experiences and see the content from a new perspective.

“*yeah it does help, it just externalizes it from yourself. You’re sort of almost then looking in on it from other people’s point of view and you also get support which is helpful”* Sparrow user.

#### “I’ve got nobody else to ask”: A last resort

For some participants, forums were a last resort rather than a first choice, which they arrived at after limited access or poor quality of NHS services. These participants described joining forums following long NHS waiting lists, a lack of support after diagnosis, or inadequate treatment options. In these situations, the forum represented an alternative sought when there were no other options.

*“What started me using them is the complete lack of information about my mental health conditions that I received from the NHS. I was given a bipolar disorder diagnosis and not given any guidance, not even a leaflet about what it was or what you had to do about it…. so the main reason I’ve been looking into forums is because I’ve got nobody else to ask.”* Starling user.

*“I’ve had a particularly difficult – I’m still in the throes of a particularly difficult time of my life. Quite a lot happened all in one go. I’ve really struggled to access decent support from my GP which is what I really needed at the time and I googled a way to get some help and be able to talk to someone”* Dunnock user.

While for some people, forums could provide a helpful alternative source of advice and support, for others it only exacerbated their feelings of distress, isolation, and disenchantment with the health system. In contrast to the previous theme where participants preferred typed communication, some participants who turned to forums as a last resort would have preferred spoken communication with health professionals instead.

*“I got to the point with a therapist on [Dunnock] she was doing my head in. She was absolutely useless and not helpful and fairly judgmental and I got to the point where it was actually doing me more harm than good using [Dunnock] and obviously I tried again with my GP to get some support and help and they were more than useless so I found a private trauma counsellor… that’s a bit more sustainable and sensible”*. Dunnock user.

#### Finding people like me: Social Connection

Participants described using forums to connect with others with similar experiences or identities, who were not accessible in their existing in-person networks. Some participants had offline friendship groups, but did not feel comfortable sharing their mental health issues because they feared being misunderstood. The forum provided a way to connect with people who understood the individual’s perspective.

*“I have a very supportive social circle but there have been moments where I have felt isolated or I have isolated myself from my social circle because I felt like they didn’t understand what I was going through… so when I see other people going through similar situations I’m motivated to talk to them”.* Chaffinch user.

One participant described specifically valuing responses on the forum from other transgender and non-binary users, whose shared lived experience meant they could understand their situation.

“*because I’m nonbinary I think I kind of appreciate the trans nonbinary people’s responses sometimes because if it’s a situation to do with how I experience dysphoria then I think sometimes that’s something that only other trans nonbinary people actually do experience in the same way*”. Jay user.

Some participants however, still struggled to find connections online, especially if they felt their experiences were uniquely challenging. For these participants, the inability to find a sense of shared experience could become a reason to stop using forums.

*“I would say [I’ve tried] maybe twenty odd forums and I find that they all kind of say the same sort of thing and I think – I know sometimes it’s changed but I think my situation is a lot more chronic than a lot of people there because they – I’ve got a lot of issues going on so my problem is quite unique I think, so I can’t resonate with – I look at people that are in them because they’ve got maybe one issue to deal with whereas I’ve got maybe five or six so it’s difficult to try and get a rapport with people like that.”.* Magpie user.

### Giving back to the community

While the previous themes described reasons for initially joining a forum, participants also described reasons for returning to forums. Some participants kept revisiting the forum even when they were no longer seeking help themselves. With more time on the forum, and more time and experience living/recovering with mental health issues, their role could shift from being a support-seeker to a support-giver, and even a moderator. They found meaning in helping newer users in similar situations and giving back to the community.

“*back when I was very unwell I used to post a lot and ask a lot of advice and share my feelings and talk to people about my feelings but now I’m feeling better and in a more stable position I tend to use the forums so I can help others and it gives me a real sense of achievement and having some use that even though it’s a stranger that I don’t know, I’ve never met and will never meet just by reaching out to them and going, ‘I’ve been in that position. This is what I did.’ I feel like I make a difference for other people and it makes me feel less useless because I feel if I can help somebody who hasn’t got help like me then there’s still a point in me being here.”*. Starling user.

This shift in role provided a sense of purpose and fulfilment that itself became a reason to continue using the forum.

### Linguistic analysis of forum posts

For a direct insight into the content of forum posts, the top ten keywords in each of the forums were examined and compared. “Scared” was the only keyword that occurred consistently across the forums, so collocations of this were explored to provide more information on the context of the word’s usage.

#### Keywords

Table 6 shows the top ten keywords from each forum corpora.

**Table 6.** Top ten keywords in each forum.

Forum	Keywords
Magpie.1	*Hi,* ***scared*** *, nt* * *, mg, depression, medication, anymore, none, hugs, mas* *
Magpie.2	***scared*** *, disorders, disorder, calories, hi, referral, nt, scary, ed* * *, helpline*
Magpie.3	*Hi, symptoms,* ***scared*** *, symptom, pregnancy, yrs, horrendous, anymore, debilitating, mums*
Magpie.4	*Hi, medication, mums, pregnancy, lithium,* ***scared*** *, scary, delusions, mg, diagnosis*
Dunnock	*nt,* ***scared*** *, hi, anymore, ur, scary, bisexual, honestly, bullied, self*
Starling	*diagnosis, referral, mental, therapy, medication, therapies, suicidal, trauma,* ***scared*** *, disorders*

^*^nt = not, mas/ed = forum related abbreviations.

The word *scared* is, based on the log likelihood statistics, the only top 10 keyword that is shared across the six datasets. The word *scared* itself occurs in the full dataset over 10,300 times, being relatively (in relation to the dataset size) the most frequent in Dunnock, as shown in Table 7. As Dunnock is, at the same time, also the biggest dataset, the following collocation analysis needs to be interpreted as primarily representing the contributions made by Dunnock users.

**Table 7.** Word count and *Number of “scared” occurrences*.

Forum	Total word count	*scared* occurrences
Dunnock	13,425,212 (48%)	7,368
Magpie	9,801,121 (35%)	2,332
Starling	4,820,253 (17%)	679
TOTAL	28,048,586	10,379

#### Collocates of “scared”

We calculated the collocates of the word *scared* to establish the major linguistic patterns of its usage across all the dataset combined. The top 60 collocates, their frequencies and log likelihood statistics values are included in Supporting Information 4; this also includes the collocates calculated for each forum separately to show the ranking and values of the collocates we analyze below.

The top collocates include many function (grammatical) words, including various alternative spellings: collocates *’m, I, im, am* all mainly occur within the phrase *I’m scared,* while prepositions and conjunctions, such as *because, of, to* indicate they may be part of phrases revealing reasons for being *scared.* We decided to look in detail at two of the top collocates: *because* and *tell. Because* (rank 4) is the first collocate that follows collocates that make up the phrase *I’m scared* (ranks 1 to 3) and *tell* is the first lexical collocate, i.e. a word with a meaning, not a grammatical word.

#### Collocate “because”: I’m scared because/because I’m scared

There is a fairly even distribution between occurrences of *because* within five words on the left and right of *scared*, which suggests that contributors both give reasons why they (or someone else) is scared and present being scared as a reason for something else.

*“because I am scared [X] will judge me”* [Dunnock]

*“I’m scared to tell [X] because I don’t want”* [Dunnock]

This collocational pair occurs in an overwhelming majority in Dunnock posts (only 6% of occurrences are found in Starling and 17% in Magpie). We further narrowed down the analysis to the phrase *scared because* (208 occurrences) as we hypothesized this may reveal reasons not only to why the participants are scared but also be relevant to wider reasons for them being on the forum. Here, the majority occurred in the first-person pronoun (87% of occurrences), with the majority occurring again in Dunnock (78%).

The qualitative analysis shows that participants share both their fears of their past experiences, fears of past experiences that are still present in their lives, and fears of current situations they find themselves in. The reasons for being scared seem to be similar across the forums, ranging from being scared of concrete things (e.g. spiders), to being worried about relationships, and very frequently of symptoms, the unknown and associated feelings:

*“Also I’m scared because they might give me medication”* [Dunnock]

*“I am scared because at night I start panicking and no longer feel in control”* [Magpie]

*“I'm quite scared because I think you're not allowed to stop taking the meds, but at the same time, if I don’t have a follow up appointment before they run out, what am I supposed to?”* [Starling]

In addition to these general themes, in Dunnock, we could identify several specific themes, which may arguably be linked to the forum’s participants’ younger age. These included themes of fear associated with school environment, both socially and in relation to academic pressure, themes of self-harm, themes of sexuality and “coming out” and relationships, including romantic relationships, more generally.

*“I want to do really well in all subjects and I’m especially scared because you have to do science GCSES and I’ve only got a grade 5”* [Dunnock]

*“I’m scared because school makes me really anxious and this is the root to so many of my issues”* [Dunnock]

*“I’ve been self-harming and I’m sort of scared because the thoughts are getting much worse, and I really don’t know what I should do”* [Dunnock]

*“It will be long before we can marry but will she still want to get married? I'm just scared because I don't want to lose her”* [Dunnock]

*“so I am gay and I want to come out to friends but im really scared because I am not sure how they will take it. does anyone have any advice”* [Dunnock]

*“So I am thinking that I might be trans and I’m scared because I have no one to talk to”* [Dunnock]

#### Collocate “tell”: Scared to tell

The collocate *tell* (495 occurrences with *scared*) is, similarly as *because,* in an overwhelming majority of occurrences (89%) linked to the Dunnock forum; this is also because it is statistically more significant among the collocates in Dunnock. More than half of these occurrences (54%) occur in a pattern where the subject is the first-person pronoun (*I*) followed by *be* verb in various forms (most frequently *am* and *was*), with several other stylistic variations with similar meaning, such as *I felt scared.*

This pattern frequently includes references to people they are *scared to tell to.* These may be general actors, such as *anyone, someone, people* (30% of occurrences) and people who are close, such as *family*, *parents,* mother and father (39%), and/or people in a position of trust, such as *friend(s)* and *teacher(s),* but, interestingly, also *doctor, nurse, therapist, councilor, CAMHS worker.* This is similar across all the forums.

While the context of occurrences is not always sufficient to determine the causes of fear, the most frequently mentioned recurring themes are broadly similar to the ones seen in the analysis of the collocate *because,* linking to sexuality (e.g. “coming out”) (21% of instances) and mental health problems and associated experiences. The description of the causes of fear is occasionally quite complex and includes cases where participants state that they suspect they may have a diagnosis which has not been confirmed yet or are scared to get a diagnosis and seek help and advice, as shown in the examples below. The data additionally contain several examples of direct requests for advice, most frequently on “how to tell” someone of their fears or offering encouragement:

*“I need an advice! Can anyone please help because I’m scared to tell my camhs worker so that they don’t think I’m self diagnosing.”* [Dunnock]

*“My parents don't know I am gay and I am scared to tell them what do I do? Any advice anyone?”* [Dunnock]

*“Don’t be scared, te*ll *the crisis support staff how you are feeling. They are there to help you. You’ll be ok*.” [Magpie]

*“But, you know, I'm really scared. Can anyone tel*l *me what their experience was like?”* [Starling]

Some of the examples explicitly mention forum participants appreciating that they can meet people they perceive as similar in the forum and that they appreciate the anonymity of the forum to freely share and find more information.

*“I have depression and [X] but I haven’t received an official diagnosis because I’m too scared to tell anyone. It’s kind of nice to see other people so similar to me.”* [Dunnock]

*“I think I have [X] but I'm not quite sure. I haven’t been diagnosed because I'm too scared to tell anyone. I was hoping with the anonymity here to get some answers.”* [Dunnock]

The majority of the posts that include this collocational pair (80%) employ “being scared to tell” in the present tense (*I am scared*), suggesting that forum participants are sharing experiences they are going through at the moment. However, there are posts where this collocational pair is part of the past tense structure, in several instances aiming to convey a positive message of “having told” and consequently feeling better, or even being articulated in the form of encouragement, see the examples.

*“I was terrified and scared to tell people. But now after having been open about it, I feel like I can finally breathe again!”* [Dunnock]

*“Hey I have been diagnosed with [X] and was really scared to tell anyone similarly like you. I told my parents two weeks ago and they were really supportive.”* [Dunnock]

*“I had a friend like this and I was scared of him but I trust in you, you should stand up to him and tell him how he makes you feel”* [Dunnock]

## Discussion

This study explored who uses online mental health forums and why, triangulating findings from a survey of 791 forum users, thematic analysis of 20 interviews, and corpus linguistic analysis of 28 million words of forum posts across seven UK-based mental health forums. Together, the findings suggest that mental health forums serve a broad population, many of whom face barriers to accessing conventional mental health support. In contrast with traditional health services, online forums provide a kind of support that is accessible, anonymous and asynchronous. The demographic comparison between forums and NHS Talking Therapies highlighted some differences with regard to the populations served. The survey indicated that forum users were mostly young (in the 16-24 age bracket), female, and White. Compared with people accessing NHS Talking Therapies, forum users included a significantly greater proportion of people aged under 25 and identifying as non-binary and as White or Mixed ethnicity. Although this may be due to over-representation of participants from Dunnock (a forum primarily aimed at young people), these findings are generally consistent with the literature that younger people are more likely to seek help online compared with older adults.^27^ However, our survey dataset was skewed by recruitment biases, as participants from Dunnock (a forum aimed at young people) contributed 287 participants out of a total of 791. The demographic heterogeneity across forums, with Magpie and Sparrow forum users mostly aged over 35, suggest that different forums may serve different populations. The heterogeneity of the forums involved is a strength of the study’s multi-forum design, although it means that the pooled survey sample is not representative of all forum users. Future research with larger per-forum samples could explore and compare forum-specific demographic profiles.

Online forums may be more appealing to gender-diverse young people compared with NHS Talking Therapies. Notably, forums attracted a higher proportion of non-binary users (5.3% non-binary compared with 0.3% recorded as “indeterminate”). The interview data supported this, as one participant valued the opportunity to connect with people with shared lived experience of gender dysphoria, connections they could not find elsewhere. Previous research has found that transphobia is a key barrier for transgender people accessing health services,^62^ because of a lack of therapists with professional knowledge or with lived experience of gender dysphoria.^63^ The linguistic analysis supported this further, as it found that fears of “coming out” to friends, family and health professionals was a common context for the word “scared” to appear in. These findings across all three methods suggest that forums play an important role for gender-diverse young people who are not able to engage with in-person health services, which is consistent with previous research showing that transgender youth value online forums because of the support networks and practical information they provide.^64^

Social connection and helping others were important reasons for forum use, as forums became spaces for peer support. 29% of survey respondents reported visiting forums to help others and interviews showed that participants wanted to connect with others with similar experiences, and that some participants transitioned from support-seekers to support-givers over time. 20% of posts including “scared to tell” were written in the past tense, with users sharing their past experiences with others. Together, these suggest that forums offer a space for peer-support, with a core userbase of returning users who find meaning and purpose in helping others. The altruistic nature of a minority of forum users has also been shown in the literature.^65,66^ For example, one study found that a small proportion (1%) of a domestic violence forum’s userbase were responsible for almost half of all posts.^67^ Having a group of people who continually return to the forum, helping others, may be a key mechanism for forums to become communities, rather than a collection of isolated posts on a common topic.^67^ Reinforcing peer support on a mental health forum is important for hosts and moderators to consider, such as by enabling virtual likes and gifts, or by moderators seeking out and thanking active users.^68^

Easy access was another reason for forum use. Survey respondents most commonly visited the forums to find help for themselves (69%) and interviews highlighted ease of account creation, access on different devices and 24/7 availability as key reasons for use. The linguistic analysis also showed that most posts sharing fears were in the present tense, suggesting users were seeking support in the moment rather than retrospectively. Together, these findings suggest that accessibility is a key enabling aspect of online forums. This is supported by previous research that found the importance of ongoing and timely access to peer support online.^22,69^ However, interviews also suggested that high accessibility could be detrimental for some users, where ongoing exposure to mental health-related content could worsen mental health. The balance between accessibility and the safety of users is important for hosts and moderators to consider when designing forums.^40^

The asynchronous and written format of forums was important to provide a space for users to share fears that they could not share elsewhere, including with NHS services. The collocation analysis of “scared” revealed that users expressed fear mostly in relation to mental health symptoms, medication, treatment, and disclosing their identity to others. These fears were shared in a written, asynchronous format which interview participants described as preferable to spoken communication, especially for participants with neurodivergence or those experiencing acute distress. The cathartic and reflective value of sharing anxieties through writing was also described, consistent with the literature on the therapeutic value of writing,^70^ and a recent review which suggested that online support groups can act as a form of narrative therapy.^71^ Importantly, forums could offer a form of support that traditional health services, which are often designed around spoken and synchronous interactions, cannot provide.

For some participants, forums were sought as a last resort after in-person services were inaccessible. This reflects the picture described in the introduction, as NHS capacity for mental health services is strained,^6^ and is consistent with previous research on forums being used by stigmatized groups who experience barriers to mental health services.^18,20^ However, forums were not always beneficial for this group. Some participants preferred professional in-person support and found the forums to be detrimental to their mental health. It may be important for forum hosts to use clear signposting for what forums can offer, and for health services to not rely on the use of forums over offering in-person or spoken support for everyone.

### Limitations

Several limitations of this study should be noted. Firstly, the survey sample was biased by Dunnock, where over a third of participants were recruited from. Therefore, the pooled forum user group was biased towards a younger demographic. Secondly, the corpus linguistic analysis was limited to three forums and a single keyword. Dunnock was also over-represented in the posts dataset, which means the findings may largely reflect the language of younger users. Future work could extend the analysis to additional keywords and forums. Another limitation of the linguistic analysis was that the BNC 1994 written component was used as reference corpus. This represents formal English from over three decades ago, which may have resulted in an artificial inflation of contractions, slang and emotional language typical of modern digital discourse. Contemporary alternatives such as Reddit-En-Base, the iWeb corpus or Glowbe could be used instead in further research, as they contain current informal, conversational online English. Finally, this study aimed to explore who uses forums and why in a UK context. It is worth noting that metadata from the Magpie posts suggested that 20% of users were based in the US, Australia, Canada and Ireland, with users from an additional 105 countries also represented. This was not investigated as part of the study’s research questions, but it indicates that findings have relevance beyond the UK context. Future research could consider the international basis of forum use more explicitly, instead of focusing on country-specific forums like in the current study and previous work.^22^

## Conclusion

Online forums for mental health are increasingly used across and beyond the UK, serving a broad population that includes many people who face barriers to in-person mental health services. Although the survey indicated the forums were used by predominantly White and female users, there is some suggestion that forums attract more ethnic diversity, more non-binary people, and younger people than NHS Talking Therapies. There is evidence that people who are questioning their gender identity or sexuality, neurodiverse people, and those with physical or geographical restrictions may feel more able to share their experiences on forums. They offer anonymous, accessible, and asynchronous spaces where people can share their fears, experience cathartic expression, connect with people with similar experiences, and give and receive support. Forums function both as a complement to NHS services, and as a last resort in their absence, forming an important addition to available mental health support. A lived experience commentary on the findings of this paper, written by a forum moderator and a service user researcher, is included in Textbox 2.

**Textbox 2. Lived experience commentary by J.S. (forum moderator) and Neil Caton**

Understanding who uses forums and why is crucial in providing insight into several key areas that can have wide-ranging impacts on forum users and the services directly or indirectly connected to them.

The mixed-methods approach has done well to capture and bring together some of the more intricate complexities involved. Some of these are not always easily recognised without the awareness of multiple perspectives, such as with the reasons behind preferences for online versus mainstream services and the ways in which these services may or may not interlink.

Moderators are in a unique position of being able to provide support much faster and to a broader range of people than many mainstream services. It is therefore encouraging to see highlighted, how forums and, by extension, their moderation have felt to be valuable, particularly for vulnerable and minority groups who may find mainstream services more inaccessible.

It is, however, important to consider that forums are being used predominantly by many of the same demographic who also use NHS Talking Therapies. This raises the question of what support is being used amongst people outside of this demographic, and whether there is a need to incorporate some of the benefits of forums and other alternative forms of support into mainstream services. This would allow forums to support a broader range of people and, if that is the case, to explore where it may be realistically possible to do so and how that could be achieved.

This research has particularly highlighted the significance of feeling connected within the support that is offered to people, but also that what constitutes feeling connected within the support is not a one-size-fits-all. This is a step in the right direction, which has the potential to lead to lasting changes that work for the current needs of a greater variety of people in a way that also provides more hope for the future, individually and collectively.

## Supplemental material

Supplemental material 1 - Who uses mental health support forums, and why? Triangulating findings from surveys, interviews, and forum posts Supplemental material for Who uses mental health support forums, and why? Triangulating findings from surveys, interviews, and forum posts by Zoe Glossop, Anna Lindroos Čermáková, Paul Marshall, Paul Rayson, Heather Robinson, Elena Semino and Fiona Lobban in Digital Health.

Supplemental material - Who uses mental health support forums, and why? Triangulating findings from surveys, interviews, and forum posts Supplemental material for Who uses mental health support forums, and why? Triangulating findings from surveys, interviews, and forum posts by Zoe Glossop, Anna Lindroos Čermáková, Paul Marshall, Paul Rayson, Heather Robinson, Elena Semino and Fiona Lobban in Digital Health.

Supplemental material - Who uses mental health support forums, and why? Triangulating findings from surveys, interviews, and forum posts Supplemental material for Who uses mental health support forums, and why? Triangulating findings from surveys, interviews, and forum posts by Zoe Glossop, Anna Lindroos Čermáková, Paul Marshall, Paul Rayson, Heather Robinson, Elena Semino and Fiona Lobban in Digital Health.

Supplemental material - Who uses mental health support forums, and why? Triangulating findings from surveys, interviews, and forum posts Supplemental material for Who uses mental health support forums, and why? Triangulating findings from surveys, interviews, and forum posts by Zoe Glossop, Anna Lindroos Čermáková, Paul Marshall, Paul Rayson, Heather Robinson, Elena Semino and Fiona Lobban in Digital Health.

## Data Availability

The anonymized survey data is openly available on Lancaster University’s PURE (https://research.lancaster-university.uk/en/datasets/improving-peer-online-forums-ipof-survey-data/). The anonymized interview transcripts are also archived on Lancaster University’s PURE and are available on request (https://research.lancaster-university.uk/en/datasets/improving-peer-online-forums-ipof-interview-data/). The forum post data is not available as participants did not consent for their data to be used outside of the study.

## References

[1] Allsopp K Kinderman P . The use of diagnoses in mental health service eligibility and exclusion criteria. Journal of Mental Health 2021; 30: 97–103. 10.1080/09638237.2019.1677875 31647342

[2] Clark DM . Implementing NICE guidelines for the psychological treatment of depression and anxiety disorders: the IAPT experience. International review of psychiatry 2011; 23: 318–327. 10.3109/09540261.2011.606803 22026487 PMC3212920

[3] Baker CK-W Esme . Mental health statistics: prevalence, services and funding in England 2023. https://web.archive.org/web/20230920144931/https://commonslibrary.parliament.uk/research-briefings/sn06988/. Accessed 30 08 2024.

[4] Punton G Dodd A McNeill A . ‘You’re on the waiting list’: An interpretive phenomenological analysis of young adults’ experiences of waiting lists within mental health services in the UK. PLOS ONE 2022; 17: e0265542. 10.1371/journal.pone.0265542 35303040 PMC8932552

[5] O’Dowd A . Hunt admits that public demand for NHS has grown beyond expectations. British Medical Journal Publishing Group, 2017. 10.1136/bmj.j5062 29092821

[6] Buivydaite R Irving D Page B , et al. Strategies for adapting under pressure: an interview study in community mental health services. Frontiers in Health Services 2025; 5: 1719583. 10.3389/frhs.2025.1719583 41459004 PMC12738820

[7] DeAndrea DC Anthony JC . Online peer support for mental health problems in the United States: 2004–2010. Psychological medicine 2013; 43: 2277–2288. 10.1017/S0033291713000172 23410539 PMC4327823

[8] Kalckreuth S Trefflich F Rummel-Kluge C . Mental health related Internet use among psychiatric patients: a cross-sectional analysis. BMC psychiatry 2014; 14: 1–11. 10.1186/s12888-014-0368-7 PMC4299476 25599722

[9] Machine IAW . Reddit r/mentalhealth. https://web.archive.org/web/20161004025537/https://www.reddit.com/r/mentalhealth/ (2023), Accessed 15th September 2025.

[10] Subreddit Stats: r/mentalhealth. https://subredditstats.com/r/mentalhealth (2023), Accessed 15th September 2025.

[11] Scottish Government . Policy Mental health 2023. https://www.gov.scot/policies/mental-health/wellbeing-and-prevention/ Accessed 29th November 2023.

[12] NHS . Support groups - Depression in adults 2023. https://www.nhs.uk/mental-health/conditions/depression-in-adults/support-groups/

[13] Togetherall . How Wandsworth CCG Alleviated Pressure on Existing Services with Togetherall. https://togetherall.com/en-gb/case-studies/taking-the-strain/ (2019), Accessed 29th November 2023.

[14] Togetherall . Going digital: How Lancashire CCGs digitise their population mental health services using Togetherall 2021. https://togetherall.com/en-gb/case-studies/helping-young-people/ Accessed 29th November 2023.

[15] NHS . Scalable innovation for mental health care and support 2023. https://www.nhsconfed.org/case-studies/scalable-innovation-mental-health-care-and-support, Accessed 29th November 2023.

[16] Bilszta J Ericksen J Buist A , et al. Women's experience of postnatal depression-beliefs and attitudes as barriers to care. Australian Journal of Advanced Nursing 2010; 27: 44–54. 10.37464/2010.273.1714

[17] McLoughlin J . Stigma associated with postnatal depression: A literature review. British Journal of Midwifery 2013; 21: 784–791. 10.12968/bjom.2013.21.11.784

[18] Moore D Ayers S . Virtual voices: social support and stigma in postnatal mental illness Internet forums. Psychology, health & medicine 2017; 22: 546–551. 10.1080/13548506.2016.1189580 27218265

[19] Kelly D Steiner A Mazzei M , et al. Filling avoid? The role of social enterprise in addressing social isolation and loneliness in rural communities. Journal of rural studies 2019; 70: 225–236. 10.1016/j.jrurstud.2019.01.024 31787802 PMC6876679

[20] Carlisle K Kamstra P Carlisle E , et al. A qualitative exploration of online forums to support resilience of rural young people in Australia. Frontiers in Public Health 2024; 12: 1335476. 10.3389/fpubh.2024.1335476 38841668 PMC11150697

[21] Smith-Merry J Goggin G Campbell A , et al. Social Connection and Online Engagement: Insights From Interviews With Users of a Mental Health Online Forum. JMIR Ment Health 2019; 6: e11084. 10.2196/11084 30912760 PMC6454344

[22] Steiner A Farmer J . Contemporary interventions tackling complex issues: Exploring pathways from online mental health forums to personal resilience. Journal of Rural Studies 2024; 110: 103379. 10.1016/j.jrurstud.2024.103379

[23] Lupton D . Young people’s use of digital health technologies in the global north: Narrative review. Journal of Medical Internet Research 2021; 23: e18286. 10.2196/18286 33427684 PMC7834940

[24] Fusar‐Poli P Correll CU Arango C , et al. Preventive psychiatry: a blueprint for improving the mental health of young people. World Psychiatry 2021; 20: 200–221. 10.1002/wps.20869 34002494 PMC8129854

[25] Salaheddin K Mason B . Identifying barriers to mental health help-seeking among young adults in the UK: a cross-sectional survey. British Journal of General Practice 2016; 66: e686–e692. 10.3399/bjgp16X687313 PMC5033305 27688518

[26] Helsper EJ Eynon R . Digital natives: where is the evidence? British educational research journal 2010; 36: 503–520. 10.1080/01411920902989227

[27] Hanley T Prescott J Gomez KU . A systematic review exploring how young people use online forums for support around mental health issues. Journal of mental health 2019; 28: 566–576. 10.1080/09638237.2019.1630725 31267813

[28] Mento C Silvestri MC Muscatello MRA , et al. Psychological impact of pro-anorexia and pro-eating disorder websites on adolescent females: A systematic review. International journal of environmental research and public health 2021; 18: 2186. 10.3390/ijerph18042186 33672305 PMC7926357

[29] Dyson MP Hartling L Shulhan J , et al. A systematic review of social media use to discuss and view deliberate self-harm acts. PloS one 2016; 11: e0155813. 10.1371/journal.pone.0155813 27191728 PMC4871432

[30] Eichenberg C . Internet message boards for suicidal people: A typology of users. CyberPsychology & Behavior 2008; 11: 107–113. 10.1089/cpb.2007.9924 18275323

[31] Mok K Jorm AF Pirkis J . The perceived impact of suicide-related internet use: A survey of young Australians who have gone online for suicide-related reasons. Digital health 2016; 2: 2055207616629862. 10.1177/2055207616629862 29942550 PMC6001192

[32] Proferes N Jones N Gilbert S , et al. Studying reddit: A systematic overview of disciplines, approaches, methods, and ethics. Social Media+ Society 2021; 7: 20563051211019004. 10.1177/20563051211019004

[33] Farmer J Steiner A Kilpatrick S , et al. Value cocreation and innovation involving consumers and providers interacting with technology: a digital ethnographic study of online mental health forums. Journal of Service Management 2025; 36: 270–290. 10.1108/josm-01-2023-0029

[34] Ammenwerth E Rigby M . Mixed methods: a paradigm for holistic evaluation of health IT. Evidence-based health informatics 2016; 102. 27198096

[35] Wilkerson DA . Lurking behavior in online psychosocial discussion forums: theoretical perspectives and implications for practice. Journal of Technology in Human Services 2016; 34: 256–266. 10.1080/15228835.2016.1193456

[36] Gries ST . A new approach to (key) keywords analysis: Using frequency, and now also dispersion. Research in Corpus Linguistics 2021; 9: 1–33. 10.32714/ricl.09.02.02

[37] Lobban F Coole M Donaldson E , et al. Improving Peer Online Forums (iPOF): protocol for a realist evaluation of peer online mental health forums to inform practice and policy. BMJ open 2023; 13: e075142. 10.1136/bmjopen-2023-075142 PMC10387651 37518092

[38] Creswell JW Clark VLP . Designing and conducting mixed methods research. Sage publications, 2017.

[39] Charalampidi M Hammond M . How do we know what is happening online? A mixed methods approach to analysing online activity. Interactive Technology and Smart Education 2016; 13: 274–288. 10.1108/itse-09-2016-0032

[40] Marshall P Caton N Glossop Z , et al. Understanding Safety in Online Mental Health Forums: Realist Evaluation. JMIR Mental Health 2025; 12: e75320. 10.2196/75320 40577699 PMC12227176

[41] Robinson H Booth M Fothergill L , et al. Understanding the Needs of Moderators in Online Mental Health Forums: Realist Synthesis and Recommendations for Support. JMIR mental health 2025; 12: e58891. 10.2196/58891 41004804 PMC12514405

[42] Shaw H Brown O Hinds J , et al. The DECIDE Framework: Describing Ethical Choices in Digital-Behavioral-Data Explorations. Advances in Methods and Practices in Psychological Science 2025; 8.

[43] Gliniecka M . The ethics of publicly available data research: A situated ethics framework for Reddit. Social Media + Society 2023; 9: 20563051231192021. 10.1177/20563051231192021

[44] Lobban F Caton N Glossop Z , et al. Evaluating Peer Online Forums to Support Health: Ethical and Practical Challenges. Journal of Medical Internet Research 2025; 27: e73427. 10.2196/73427 41442282 PMC12732582

[45] Harris PA Taylor R Minor BL , et al. The REDCap consortium: building an international community of software platform partners. Journal of biomedical informatics 2019; 95: 103208. 10.1016/j.jbi.2019.103208 31078660 PMC7254481

[46] Westhorp G Feeny S . Using surveys in realist evaluation. Evaluation Journal of Australasia 2025; 25: 45–64. 10.1177/1035719x241292083

[47] Lobban F Coole M Donaldson E , et al. Improving Peer Online Forums (iPOF) Survey Data, 2025.

[48] NHS . ​NHS Talking Therapies, for anxiety and depression. Annual reports 2024; 2022-23. https://digital.nhs.uk/data-and-information/publications/statistical/nhs-talking-therapies-for-anxiety-and-depression-annual-reports/2022-23, Accessed 30th August 2024.

[49] Corporation M . Microsoft Excel, 2024.

[50] McHugh ML . The chi-square test of independence. Biochemia medica 2013; 23: 143–149. 10.11613/bm.2013.018 23894860 PMC3900058

[51] Marshall P Booth M Coole M , et al. Understanding the Impacts of Online Mental Health Peer Support Forums: Realist Synthesis. JMIR Ment Health 2024; 11: e55750, Original Paper 9.5.2024. 10.2196/55750 38722680 PMC11117133

[52] Clarke V Braun V . Thematic analysis. The Journal of Positive Psychology 2016; 12(3): 297–298. doi:10.1080/17439760.2016.1262613. https://www.tandfonline.com/doi/full/10.1080/17439760.2016.1262613

[53] Dhakal K . NVivo. Journal of the Medical Library Association: JMLA 2022; 110: 270. 10.5195/jmla.2022.1271 35440911 PMC9014916

[54] Seale C Ziebland S Charteris-Black J . Gender, cancer experience and internet use: a comparative keyword analysis of interviews and online cancer support groups. Social science & medicine 2006; 62: 2577–2590. 10.1016/j.socscimed.2005.11.016 16361029

[55] Semino E Baker P Brookes G , et al. Applying Corpus Linguistics to Illness and Healthcare​. ​Cambridge University Press, 2025.

[56] Consortium BNC . British National Corpus Sampler, 2007. https://llds.ling-phil.ox.ac.uk/llds/xmlui/handle/20.500.14106/2551

[57] Consortium BNC . British national corpus. The BNC Consortium, 2007. https://www.natcorp.ox.ac.uk

[58] Dunning T . Accurate methods for the statistics of surprise and coincidence. Computational linguistics 1994; 19: 61–74.

[59] Sinclair J . Corpus, concordance, collocation, 1991.

[60] Anthony L . AntConc. Waseda University, 2014. [Computer Software].Version 3.4.3.

[61] IBM . Cramer's V 2024. https://www.ibm.com/docs/en/cognos-analytics/11.1.0?topic=terms-cramrs-v, Accessed 17th January 2025

[62] Safer JD Coleman E Feldman J , et al. Barriers to healthcare for transgender individuals. Current Opinion in Endocrinology, Diabetes and Obesity 2016; 23: 168–171. 10.1097/MED.0000000000000227 26910276 PMC4802845

[63] Hayes E . Why Would They Listen to You? You’re Just a Crazy Trans Person”-Understanding the Experiences of the Trans Community When Accessing NHS Mental Health Services: A Thematic Analysis. University of East London, 2025.

[64] Evans YN Gridley SJ Crouch J , et al. Understanding online resource use by transgender youth and caregivers: A qualitative study. Transgender health 2017; 2: 129–139. 10.1089/trgh.2017.0011 29082333 PMC5628561

[65] Rathbone AL Clarry L Prescott J , et al. Digital altruism: the motivators for, effects of and support received when moderating mental health online. Mental Health and Digital Technologies 2024; 1: 37–52. 10.1108/mhdt-12-2023-0004

[66] Ros-Galvez A Rosa-Garcia A . Private provision of a public good: cooperation and altruism of internet forum users. International Journal of the Commons 2015; 9: 720–743. 10.18352/bmgn-lchr.554

[67] Lindgren S . Giving online support: Individual and social processes in a domestic violence forum. International journal of web based communities 2014; 10: 147–157. 10.1504/ijwbc.2014.060352

[68] Marshall P Caton N Cermakova AL , et al. Designing online forums to support health and wellbeing: guidance informed by self-determination theory and stakeholder perspectives. JMIR Preprints 2025. 10.2196/preprints.85411

[69] Prescott J Rathbone AL Hanley T . Online mental health communities, self-efficacy and transition to further support. Mental Health Review Journal 2020; 25: 329–344. 10.1108/mhrj-12-2019-0048

[70] Nagel D Anthony K . Writing therapy using new technologies—The art of blogging. Journal of poetry therapy 2009; 22: 41–45. 10.1080/08893670802708001

[71] Santo C dos Santos Silva I Soares L . Scientific Evidence Supporting Narrative Therapy and Therapeutic Writing in Treating Diseases—A Literature Review about Online Support Groups. Creative Therapeutic 2025; 1: 35–52. 10.54963/ct.v1i1.1712

