# Supplementary material for: Who uses mental health support forums, and why? Triangulating findings from surveys, interviews, and forum posts
Source: Digit Health. 2026 Jun 5;12:20552076261458957. doi: 10.1177/20552076261458957 (PMC13241684; doi:10.1177/20552076261458957)
Supplement: Supplemental material 1 - Who uses mental health support forums, and why? Triangulating findings from surveys, interviews, and forum posts [file sj-pdf-1-dhj-10.1177_20552076261458957.pdf]

Supporting Information 1 – Survey Items and Coding

Question	Response Options
“Thinking about the **first time** you ever came to this forum, which options best describe **the main reason** for your visit?” (pick more than one if you like)	I wanted to find help, advice, information, or support for **myself** (yes = 1, no = 0)<br>I wanted to find help, advice, information, or support for **someone else** (e.g. friends, family) (yes = 1, no = 0)<br>I wanted to offer help, advice, support, or information to **other forum users**<br>(yes = 1, no = 0)<br>Other reasons (please write in)”<br>(yes = 1, no = 0))
“What is your gender? (Select one)”	0 “female”<br>1 “male”<br>2 “non-binary”<br>3 “prefer not to say”<br>4 “prefer to self-describe (please write in)”
“What is your age group? (Select one)”	0 “16 – 24”<br>1 “25 – 34”<br>2 “35 – 44”<br>3 “45 – 54”<br>4 “55 -- 64”<br>5 “65 and over”<br>6 “prefer not to say”
“How would you describe your ethnicity? (Select one)”	0 “White”<br>1 “Black”<br>2 “Asian”<br>3 “Mixed”<br>4 “prefer not to say”<br>5 “prefer to self-describe (please write in)”

To ensure categories were comparable across the survey and Talking Therapies, the following categories from the survey were recoded. For gender, “Non-binary” and “prefer to self-describe” were recoded into “Indeterminate”. For ethnicity, “prefer to self-describe” was recoded into “other ethnic group”. For age, “16 – 24” was recoded into “Under 25” and all age groups between 25 and 64 were recoded into “25 – 64”.
