# Supplementary material for: Who uses mental health support forums, and why? Triangulating findings from surveys, interviews, and forum posts
Source: Digit Health. 2026 Jun 5;12:20552076261458957. doi: 10.1177/20552076261458957 (PMC13241684; doi:10.1177/20552076261458957)
Supplement: Supplemental material - Who uses mental health support forums, and why? Triangulating findings from surveys, interviews, and forum posts [file sj-pdf-3-dhj-10.1177_20552076261458957.pdf]

Supporting Information 3 – Additional Quotes from Thematic Analysis

Supplementary table 2: Additional quotes	
Theme Title	Quote
Anytime, Anywhere: The Value of Accessibility	"*I think I take more from the forum than I give because I mostly just read things"* - Robin user.
	"It is easily available and it was just referred by a friend and I got to use it easily so I think the availability played a very big role". – Jay user
	"*I suppose you just kind of scroll out – like normally on [Starling] I suppose like mindlessly scrolling to a degree then you see a post that may resonate with you".* Starling user
	"*I have come across posts as well which are quite actually concerning as well in terms of the content, you know like someone's going through a sensitive issue, very kind of – it is quite horrifying to be honest, it could happen to anyone. Makes you concerned that – can make you paranoid that it can happen to you perhaps*" Starling user.
"Happier to Type": In Your Own Time, Space and Words	"*people in my situation [*with head injuries*] don't seem very good at interacting socially… in person somehow it's much harder"* Magpie user.
	"lots of people that really don't like phone calls and obviously typing it's a lot easier". Starling user.
	"*unfortunately I've lost quite a lot of my hearing… if there's more than one conversation going on nearby I can't pick out what somebody is actually saying to me because the conversation is just a conversation*". Magpie user
	"I struggle to talk to anyone face to face about my issues… I don't like getting emotional in front of people, so through an anonymous website that's much easier to do because you're through a computer screen so it's much less stressful" Dunnock user
	"*I first came to join Robin because the bipolar support group was suggested to me by the psychiatrist and I wasn't in the right frame of mind to do a group but I looked up online and saw that they had a forum so I joined the forum straight away*" Robin user.
	"*back when I was very unwell I used to post a lot and ask a*

	lot of advice and share my feelings and talk to people about my feelings". Starling user.
Finding People Like Me: Social Connection	*It's nice to know there's somewhere I can go if I'm feeling down or whatever. I would say that the besides (Dunnock) I don't really have that in my actual life" Dunnock user.*
	"Just communication I think really because I don't go out an awful lot and I don't see a lot of people so it's nice to communicate and with people perhaps in the same position who have been through depression and anxiety and that sort of thing really". Sparrow user.
	"I wanted to see what other people's experiences were of bipolar disorder and just feel not alone with it…I think when you go and see healthcare professionals they only know about the disorder from the scientific point of view but they don't really know how it is to live with it" Robin user.
Giving Back to the Community	*"when I see other people going through similar situations I'm motivated to talk to them and there's just that feeling of relatability and just thinking, 'Oh my god this person is going through the same thing,' or some people have questions and I think, 'Oh my god, I understand what they're going through.' How can I help them and ensure that they're okay and they don't end up in the situation that I have ended up in the past, so it's like trying to comfort them, trying to support them"* Chaffinch user.
	"*Yeah, it's nice to be able to provide people with things that you wish you could have had yourself or even things that you wish you could have now. I've noticed myself in a lot of users like everyone is really good on the sub at making long posts and adding resources for things that they've personally looked up and there's very much a vibe that everyone wants to be there for each other.*" Starling user.
