# Supplementary material for: Who uses mental health support forums, and why? Triangulating findings from surveys, interviews, and forum posts
Source: Digit Health. 2026 Jun 5;12:20552076261458957. doi: 10.1177/20552076261458957 (PMC13241684; doi:10.1177/20552076261458957)
Supplement: Supplemental material - Who uses mental health support forums, and why? Triangulating findings from surveys, interviews, and forum posts [file sj-pdf-4-dhj-10.1177_20552076261458957.pdf]

Supporting Information 4 – Table of top 60 collocations of “scared”

The full dataset				Dunnock				Magpie				Starling*			
**Collocate**	**Rank**	**Freq**	**Likelihood**	**Collocate**	**Rank**	**Freq**	**Likelihood**	**Collocate**	**Rank**	**Freq**	**Likelihood**	**Collocate**	**Rank**	**Freq**	**Likelihood**
m	1	4151	10402.341	m	1	2991	6745.246	m	1	805	1990.235	m	1	355	1239.08
i	2	10005	5917.406	i	2	7013	3118.909	i	2	2239	1294.073	i	2	753	1047.49
im	3	1000	2814.602	im	3	849	1898.164	am	3	319	446.904	too	3	66	174.877
**because**	4	1142	1762.251	of	4	1766	1208.114	im	4	131	417.735	so	4	114	135.885
too	5	955	1687.26	too	5	703	1185.941	of	5	650	357.753	**because**	5	62	124.571
am	6	1074	1484.263	**because**	6	889	1068.307	so	6	432	336.453	of	6	199	97.593
of	7	2615	1426.09	am	7	697	1051.905	**because**	7	191	327.732	you	8	40	80.953
so	8	1632	1035.76	to	8	3441	677.945	too	8	186	267.165	am	9	58	78.161
but	9	1865	878.465	was	9	794	639.613	health	9	19	257.143	was	10	92	74.943
you	10	1171	826.854	you	10	884	613.682	you	10	247	228.326	im	11	20	71.371
was	11	1217	764.105	so	11	1086	521.326	support	11	42	193.256	really	12	48	67.561
a	12	797	731.556	but	12	1391	507.299	scared	12	62	192.729	anxious	13	16	63.186
**tell**	13	500	709.756	a	13	526	485.108	but	13	376	183.353	going	14	37	60.942
to	14	4599	616.898	**tell**	14	444	477.153	was	14	331	183.041	ll	15	33	57.074
health	15	47	572.756	going	15	379	338.64	mental	15	45	154.163	go	16	36	56.511
going	16	521	500.959	be	16	937	328.668	a	16	200	148.477	but	17	98	50.061
support	17	56	498.807	will	17	506	285.327	your	17	55	137.642	for	18	36	47.521
mental	18	66	476.847	really	18	575	235.96	feel	18	191	106.053	health	19	8	46.281
really	19	753	464.759	can	19	171	223.723	incase	19	14	90.562	now	20	35	45.601
scared	20	220	436.519	scared	20	158	218.724	being	20	101	89.591	mental	21	8	45.464
ll	21	315	343.868	incase	21	45	210.832	losing	21	27	88.646	hurt	22	10	42.449
incase	22	60	316.083	ll	22	231	206.445	death	22	26	84.655	your	23	4	42.281
can	23	252	305.744	gonna	23	96	196.005	anxious	23	36	82.104	that	25	119	35.222
https	24	1	303.108	nervous	24	74	167.619	going	24	105	78.524	a	26	71	34.481
anxious	25	141	282.529	happen	25	96	161.904	part	25	112	78.371	what	27	49	34.273
being	26	386	268.304	with	26	227	160.85	x	26	24	77.238	me	28	69	32.925
on	27	287	258.678	ask	27	166	153.27	psychosis	27	6	75.074	confused	29	7	32.688
your	28	332	252.359	being	28	256	149.762	on	28	92	71.891	she	30	31	31.679
gonna	29	105	248.946	and	29	2663	143.902	really	29	130	71.6	here	31	7	31.479
will	30	656	243.569	get	30	489	143.19	just	30	185	66.694	support	32	5	31.022
the	31	1424	235.92	anxious	31	89	137.918	me	31	261	63.583	uk	33	1	29.834
with	32	337	235.333	parents	32	193	135.245	good	32	11	62.039	can	34	17	28.813
be	33	1240	226.999	hope	33	31	125.625	have	33	120	61.713	being	35	29	28.311
part	34	125	221.952	have	34	338	123.236	go	34	99	60.971	**tell**	36	14	25.647
happen	35	124	215.292	your	35	273	108.614	care	35	3	59.719	needles	37	3	24.953
nervous	36	83	213.72	yourself	36	24	103.933	in	36	119	58.348	on	38	15	24.864
in	37	488	213.262	this	37	268	99.996	ll	37	51	58.112	and	39	261	24.835

and	38	3719	210.139	about	38	549	99.622	worried	38	28	58.095	my	40	79	24.524
go	39	405	208.231	in	39	341	96.852	action	39	8	56.292	the	41	114	24.414
x	40	34	201.33	that	40	1099	96.833	about	40	156	56.105	in	42	28	24.315
have	41	489	199.054	worried	41	81	95.282	alone	41	48	55.252	bit	43	16	24.311
parents	42	203	197.889	the	42	899	95.19	with	42	88	53.511	vaccine	44	4	23.59
about	43	756	196.62	anyone	43	206	94.906	feeling	43	81	51.939	just	45	44	21.874
me	44	1128	188.983	on	44	180	94.705	again	44	73	51.676	embarrassed	46	4	21.428
worried	45	114	178.751	won	45	97	92.832	hi	45	7	51.637	from	47	4	20.654
ask	46	194	174.158	lose	46	48	90.922	dying	46	13	48.892	dark	48	2	20
for	47	603	164.31	people	47	401	90.159	to	47	888	47.603	leave	49	9	19.695
that	48	1550	160.356	needles	48	18	88.569	can	48	64	44.122	who	50	5	19.641
get	49	613	156.05	go	49	270	85.842	frightened	49	10	42.643	muslims	51	2	17.794
losing	50	61	150.197	leave	50	84	84.837	hope	50	9	42.133	an	52	5	17.73
death	51	57	146.313	helps	51	12	81.731	for	51	142	41.954	found	53	1	17.385
good	52	60	144.41	good	52	47	79.827	be	52	237	38.71	abandoned	54	3	17.015
hope	53	43	142.589	might	53	164	79.575	**tell**	53	42	38.563	him	55	13	16.816
feel	54	804	142.553	rejection	54	18	73.51	there	54	26	37.574	inexperienced	56	2	16.781
leave	55	117	140.487	x	55	10	73.108	happen	55	24	37.277	happening	57	6	16.707
just	56	785	132.031	react	56	32	72.069	lonely	56	17	36.91	outside	58	7	16.571
action	57	9	131.798	judge	57	39	71.319	paranoid	57	11	36.274				
uk	58	2	130.459	love	58	44	70.795	and	58	795	34.126				
care	59	15	128.006	like	59	311	70.636	from	59	27	29.845				
from	60	92	124.925	my	60	1028	69.635	some	60	19	29.665				

*Starling had 56 collocates of “scared”, hence less than 60 are displayed.
